# Protective potential of dimethyl fumarate in a mouse model of thalamocortical demyelination

**DOI:** 10.1007/s00429-018-1680-7

**Published:** 2018-05-09

**Authors:** Manuela Cerina, Venu Narayanan, Anna Delank, Patrick Meuth, Stephanie Graebenitz, Kerstin Göbel, Alexander M. Herrmann, Stefanie Albrecht, Thiemo Daldrup, Thomas Seidenbecher, Ali Gorji, Tanja Kuhlmann, Heinz Wiendl, Christoph Kleinschnitz, Erwin J. Speckmann, Hans-Christian Pape, Sven G. Meuth, Thomas Budde

**Affiliations:** 1 Department of Neurology with Institute of Translational Neurology, Westfälische Wilhelms-University, Mendelstrasse 7, 48149 Münster, Germany; 2 Institute of Physiology I, Westfälische Wilhelms-University, Robert-Koch-Str. 27a, 48149 Münster, Germany; 3 Institute of Neuropathology, University Hospital Münster, Münster, Germany; 4 Epilepsy Research Center, Westfälische Wilhelms-University, Münster, Germany; 5 Department of Neurology, Essen University Hospital, Essen, Germany

**Keywords:** Multiple sclerosis, Voltage-sensitive dye, Demyelination, Remyelination, Thalamocortical system, Auditory cortex, Spatiotemporal activity pattern

## Abstract

Alterations in cortical cellular organization, network functionality, as well as cognitive and locomotor deficits were recently suggested to be pathological hallmarks in multiple sclerosis and corresponding animal models as they might occur following demyelination. To investigate functional changes following demyelination in a well-defined, topographically organized neuronal network, in vitro and in vivo, we focused on the primary auditory cortex (A1) of mice in the cuprizone model of general de- and remyelination. Following myelin loss in this model system, the spatiotemporal propagation of incoming stimuli in A1 was altered and the hierarchical activation of supra- and infragranular cortical layers was lost suggesting a profound effect exerted on neuronal network level. In addition, the response latency in field potential recordings and voltage-sensitive dye imaging was increased following demyelination. These alterations were accompanied by a loss of auditory discrimination abilities in freely behaving animals, a reduction of the nuclear factor-erythroid 2-related factor-2 (Nrf-2) protein in the nucleus in histological staining and persisted during remyelination. To find new strategies to restore demyelination-induced network alteration in addition to the ongoing remyelination, we tested the cytoprotective potential of dimethyl fumarate (DMF). Therapeutic treatment with DMF during remyelination significantly modified spatiotemporal stimulus propagation in the cortex, reduced the cognitive impairment, and prevented the demyelination-induced decrease in nuclear Nrf-2. These results indicate the involvement of anti-oxidative mechanisms in regulating spatiotemporal cortical response pattern following changes in myelination and point to DMF as therapeutic compound for intervention.

## Introduction

Alterations in cortical layering, cellular organization, and functionality were recently identified as pathological hallmarks characterizing diseases like multiple sclerosis (MS) following demyelinating and inflammatory lesion formation (He et al. 2009; Deppe et al. 2014). Time points and location of lesioning define the symptoms and deficits that can be observed both in patients and animal models (Dubois-Dalcq et al. 2005; Crawford et al. 2009b; Rodgers et al. 2013; Deshmukh et al. 2013; Cerina et al. 2017). Thus, visual deficits may occur following damage to the visual cortex or locomotor impairment following alteration of the motor cortex (Vitorino et al. 2016). Recent findings obtained from structural and functional MRI studies in MS patients pointed out that altered memory, cognition, or locomotion was associated not only with grey matter damage in terms of atrophy but was accompanied by a profound reorganization of network topology. After all, cortical reorganization or alteration certainly depends on mechanisms to adapt and respond to pathologic conditions (Ziskind-Conhaim and Redman 2005; Gamboa et al. 2014). In MS patients, reorganization was suggested to be triggered by an altered degree of demyelination at different stages of the disease (He et al. 2009). Disappearance of myelin, changes in axonal thickness, and the tightness of wrapping myelin sheets can result in altered excitability, modified stimulus propagation, and cell death (Nave and Werner 2014; Calabrese et al. 2015), with important consequences for neuronal network functionality and topology (Crawford et al. 2009a; Ehling et al. 2011; Hamada and Kole 2015; Cerina et al. 2017).

However, the mechanisms underlying these changes in network reorganization are not well understood. Therefore, we aimed to analyze the structural and functional consequences of cuprizone-induced de- and remyelination on the properties of the auditory cortical neuronal network as a model system which reveals well-defined anatomical and functional characteristics (Matsushima and Morell 2001; Skripuletz et al. 2011). By adding the copper chelator cuprizone to the diet, we induced oligodendrocyte death and, therefore, demyelination. Removal of the compound from the diet results in remyelination (Skripuletz et al. 2011) mimicking relevant aspects of the human disease. By means of voltage-sensitive dye (VSD) imaging and extracellular field potential recordings, we observed changes in neuronal network properties in the primary auditory cortex in mice characterized by altered spatiotemporal propagation of the stimulus within A1 layers following general myelin loss. Interestingly, these functional changes in cortical network behavior persisted during remyelination. These results suggest that strategies of remyelination might be combined with (neuro)protective approaches, while critical time windows for therapeutic interventions have to be defined. Here, we tested the effects of dimethyl fumarate (DMF) during remyelination. DMF is a fumaric acid derivate which was proven to be effective as a cytoprotective drug by promoting viability of neurons, astrocytes and oligodendrocytes in a number of studies in vitro and in different animal models (Linker et al. 2011; Scannevin et al. 2012; Bomprezzi 2015; Tambalo et al. 2015). Nowadays, this compound is approved for the treatment of relapsing-remitting MS, but its exact mechanism of action remains unclear. The positive effects are believed to be exerted by its metabolite monomethyl fumarate (MMF) on the regulation of the Nrf-2 pathway. The latter is a nuclear factor whose activation triggers the transcription of genes involved in the anti-oxidative stress response, thereby restricting cell death (Fox et al. 2014; Reick et al. 2014). In our study, therapeutic treatment with DMF during remyelination resulted in the prevention of remyelination-induced loss of nuclear Nrf-2 and ameliorated cortical functionality both in vitro and in vivo.

## Materials and methods

### Animals and experimental design

All work performed on animals, in vitro and ex vivo, was performed according to the 2010/63/EU of the European Parliament and of the Council of 22 September 2010 and has been approved by local authorities (Landesamt für Natur, Umwelt und Verbraucherschutz Nordrhein-Westfalen; approval IDs: 87-51.04.2010.A331 and 84-02.04.2015.A585). All efforts were made to minimize the number of animals used and to avoid their stress and suffering by strictly following the ARRIVE guidelines (Kilkenny et al. 2010). C57BL6J mice were used for all experiments, were group-caged, and kept in a 12-h light/dark cycle. Food and water were available *ad libitum*.

#### Cuprizone treatment

**Fig. 1:**
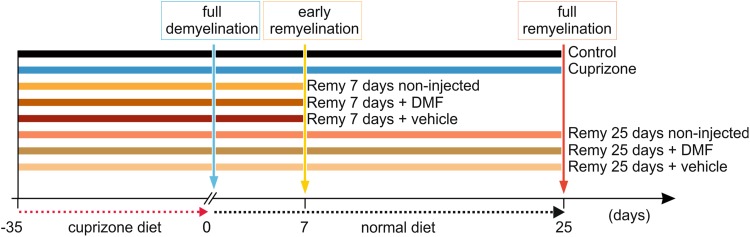
Schematic representation of the experimental outline. The horizontal black arrow indicates the time line starting with the beginning of the cuprizone diet, which lasted for 35 days (5 weeks; red arrow). At this point, the animals were re-introduced to normal food till the end of the experiments. The different experimental groups were tested in vitro and in vivo, at the end of the cuprizone diet (full demyelination—blue inset) and at the early (yellow inset) and late phases (orange inset) of remyelination, namely 7 and 25 days after re-introduction of normal food. In addition, the horizontal color-coded lines indicated the experimental outline for the treatment with DMF. Treatment started when normal food was re-introduced to the diet and it lasted for 7 or 25 days. The group of DMF-treated animals was compared to vehicle-treated or non-injected mice, which underwent the same handling

General experimental toxic demyelination was induced by feeding one group of mice (8–12 weeks of age at the beginning of the experiment) and a diet containing 0.2% cuprizone (bis-cyclohexanone oxaldihydrazone, Sigma–Aldrich Inc., Hamburg, Germany) mixed into a ground standard rodent chow (Skripuletz et al. 2011). The cuprizone diet was maintained for 5 weeks (Fig. 1). A second group of animals, matched for age and sex, served as a control. Interruption of the cuprizone diet promotes spontaneous remyelination (Skripuletz et al. 2008); therefore, we included two additional groups in our study that were investigated 7 and 25 days after re-introduction of normal food (referred to as remyelination 7 days and remyelination 25 days in the text; Cerina et al. 2017 and Fig. 1). The experiments were repeated twice.

#### Dimethyl fumarate treatment

Six additional groups of mice were used for the experiments involving dimethyl fumarate (DMF; Tocris, Bio-Technology, Germany). After treatment with cuprizone (see above), two groups of ten animals each were treated daily with an intraperitoneal injection of 15 mg/kg DMF diluted in NaCl (0.9%; Reick et al. 2014) for 7 and 25 days followed by ex vivo analysis. Two additional groups matched for age and gender were used as controls receiving no injections or injected with vehicle (Fig. 1). The experiments were repeated twice.

### Preparation of brain slices

Brain slices containing the auditory thalamocortical system, which includes the primary auditory cortex (A1), the thalamic medial geniculate nucleus (MGN), and the functional thalamocortical projection (internal capsule—IC), were obtained as described previously (Broicher et al. 2010). Briefly, mice (8–12 weeks of age) were deeply anesthetized with isoflurane (4% in O_2_) and decapitated. Brains were quickly removed and glued with the dorsal side to a 25° agar ramp block which was placed on a vibratome (Leica, Germany) and superfused with ice-cold artificial cerebrospinal fluid (ACSF) containing the following in mM: sucrose, 200; glucose, 10; PIPES, 20; KCl, 2.5; MgSO_4_, 10; CaCl_2_, 0.5; pH 7.35 with NaOH; sagittal angled slices were then cut (500 µm). Slices were incubated with the voltage-sensitive dye RH-795 (Invitrogen, Karlsruhe, Germany; 12 µg/ml, dissolved in oxygenated ACSF) at 31 °C for 60 min. Thereafter, slices were transferred to a holding chamber (oxygenated ACSF, 31 °C) and allowed to rest for 60 min to remove the additional dye, before the experiments started. The `*n`* numbers stated in the text refer to the number of slices recorded and analyzed.

### Recording of voltage-sensitive dye and electrical signals

**Fig. 2:**
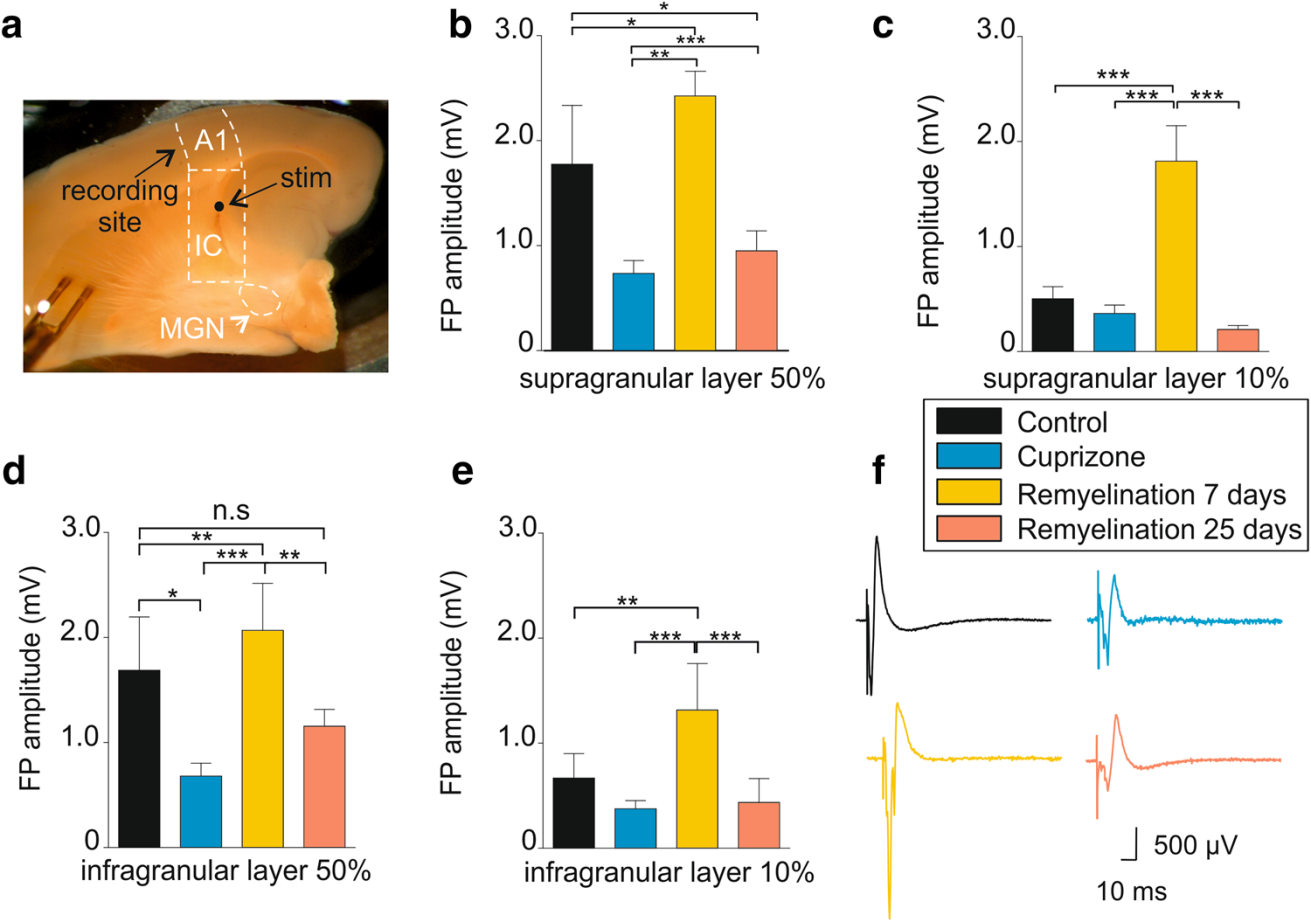
Electrophysiological analysis of the auditory thalamocortical system during de- and remyelination. **a** Micrograph shows the preserved auditory thalamocortical pathway in acute brain slice with auditory thalamus (MGN), internal capsule (IC), and primary auditory cortex (A1). The position of electrical stimulation (stim) is indicated. **b, c** Bar graphs showing the change in the LFP amplitude in the four experimental groups for 50 and 10% stimulus intensities in the supragranular layer. **d, e** Bar graphs showing the change in the LFP amplitude in the four experimental groups for 50% and 10% stimulus intensities in the infragranular layer. **f** Example traces showing the LFP components for all experimental groups in response to 50% SI. *p < 0.05, **p < 0.01, ***p < 0.001

Optical and electrical signals were simultaneously recorded from slices kept at ~ 30 °C in a submerged recording chamber mounted on an inverted microscope (Zeiss, Göttingen, Germany). Optical recordings were performed in A1 as described previously (Broicher et al. 2010) and governed by the software Neuroplex (Redshirt Imaging, Decatur, GA, USA). Fluorescence changes were detected using a hexagonal photodiode array composed of 464 elements through a 20× objective detecting an area of 0.416 mm^2^ which covers all the layers of the auditory cortex in a given slice (Fig. 4a). The sampling interval was 1.274 ms and the maximal length of the recording was 1305 ms (maximal exposure time to the Xenon lamp). Three repeated detections with an interval of 10 s were averaged to increase the signal quality and to improve the signal­to­noise ratio. There was an interval of 5 min between each averaged detection event to allow slices to recover and ensure that baseline values were reached after acquisition. The excitation wavelength of RH 795 was bandpass filtered at 546 ± 20 nm, and after passing a dichroic mirror, emitted light was high-pass filtered at 590 nm with transmission and emission maxima being 530 and 712 nm, respectively. Optical signals were recorded in parallel with electric signals induced by a custom-made bipolar electrode placed in the thalamocortical projections in a rostral or caudal position in respect to the cortex (Figs. 2a, 4a). Stimulation was triggered by the software Axoscope (Molecular Devices, Sunnyvale, CA, USA) and responses were detected by two recording electrodes (filled with ACSF; *R*_pipette_, 0.8–1.5 MΩ) positioned in the supragranular and infragranular layers of the auditory cortex. Pulse width for individual stimulation (~ 1 mA, 100%) and stimulation length (150 µs) were set to obtain optimal responses. If not indicated otherwise, the stimulation strength was set to 50% throughout the experiment.

### Analysis of optical and electrical signals

Functionally, the layers of the cerebral cortex can be roughly divided into three parts: The supragranular layers consist of layers I to III (origin and termination of intracortical connections), the granular layer IV (receives thalamocortical connections), and the infragranular layers consist of layers V and VI [primarily connecting the cerebral cortex with subcortical regions (Winkowski and Kanold 2013)]. To account for this different functional pattern and to analyze the laminar activation profile, we grouped cortical layers in A1 as supragranular (I, II, and III) and infragranular (IV, V, and VI) layers (Broicher et al. 2010; O’Connell et al. 2014; Atencio et al. 2016). Layer IV was included to the infragranular layers, since the initial response in this layer following electrical stimulation was rather distinct and short lasting (cf. Fig. 4g, left panel, frame 128) and similar to layer V and VI thereafter. Amplitude and latency of responses to electrical stimulation were taken as read-out parameters and calculated by averaging six adjacent diodes acquired in three consecutive trials for the two cortical regions grouped together (supragranular: layers I, II, and III; infragranular: layers IV, V, and VI).

**Fig. 3:**
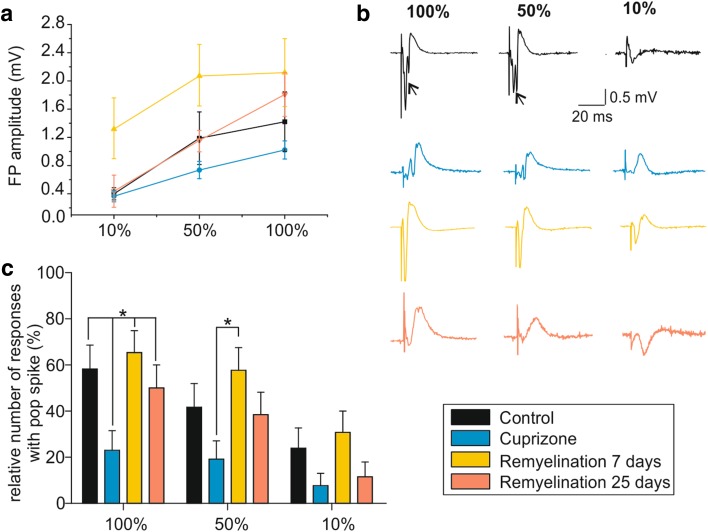
Basic neuronal response changes following de- and remyelination. **a** Extracellular recordings of local FP in response to increasing stimulus intensities (10, 50, and 100% when 50% ~ 500 µA) were used to generate input/output curves. **b** Example traces showing LFP components for all experimental groups and the three different stimulation intensities. Arrows indicate population spikes (i.e., the summed action potentials (APs) of many neurons). **c** Bar graph showing the difference in the threshold for generation of a population spike in the different groups. For each SI, the presence or absence of population spikes in the LFP was determined and expressed as a percentage of the total number of slices analyzed. *p < 0.05

Optical signals were expressed as fractional changes of the fluorescence from the resting light intensity (*I*_rest_ − *I*_recording_/*I*_rest_; d*I*/*I* in the text). Data analysis was performed manually using Neuroplex (Redshirt Imaging, USA) and custom-made MATLAB scripts. Amplitude values are given as peak amplitude minus baseline (mean amplitude of the 50 ms period preceding the stimulus); latency was determined as the time between the stimulus application and the peak of the response (Supplementary Fig. 1a). For construction of spatiotemporal cortical inputs, data were converted to fluorescence pseudo-colored maps and scaled to the maximal signal of the recording session. Fluorescence amplitudes of infragranular and supragranular cortical layers were compared between the experimental groups and normalized to control (d*I*/*I*, 0.34%) to allow direct comparison of the changes in fluorescence intensity. The field potential amplitude simultaneously recorded with the optical signals was analyzed as distance in µV from the most negative to the most positive peak in the recordings (Supplementary Fig. 1b). The relative number of stimulations applied to a given slice which triggered a population spike (indicated by the arrow in Supplementary Fig. 1b) with different stimulation strengths (10, 50, and 100%) was determined (Fig. 3c). Return of the voltage signal to baseline was assessed 25 ms after stimulus application. The *n* in the text is given as number of slices recorded and analyzed.

### Behavioral testing

Different behavioral tests were performed using cuprizone-treated animals (at remyelination day 7 and 25), treated with DMF or vehicle compared to respective controls as specified in the text.

#### Auditory Pavlovian conditioning

A modified fear conditioning paradigm was performed as described previously (Narayanan et al. 2011; Daldrup et al. 2015; Cerina et al. 2017). Mice were adapted twice per day (6 h delay) to the fear conditioning apparatus (TSE System GmbH, Bad Homburg, Germany) while being exposed to six neutral tones (unconditioned stimulus CS^−^, 2.5-kHz tone, 85 dB, 10-s duration; non-relevant stimulus in the text). On the next day, animals were exposed to the conditioned stimulus (three trials; CS^+^, 10-kHz tone, 85 dB, 9-s duration; conditioned stimulus in the text) randomly coupled with a mild foot-shock (0.4 mA, 1-s duration, onset with CS termination). After 24 h from the last tone presentation, freezing was taken as behavioral read-out, namely the duration of immobility (except respiration movements) in the animal in response to the presentation of the conditioned stimulus (10 kHz), as described previously (Narayanan et al. 2011; Daldrup et al. 2015; Cerina et al. 2017) and schematically represented in Fig. 7a.

#### Locomotor activity and anxiety-like behavior

Animals were tested using the Elevated Plus Maze (EPM, Ethovision, Noldus IT bv, Wageningen, The Netherlands) to assess anxiety-like behavior. Each group consisting of five animals underwent 5 min of exploration on the testing device, while the time spent in closed and open arms was taken as read-out. The same animals were tested in the Open Field arena (35 × 40 × 40 cm) after an interval of at least 5 h from undergoing the EPM, to assess locomotor activity and the travelled distance; the number of crossings and vertical exploratory behavior was taken as a read-out (Noldus Ethovision, The Netherlands).

### Histological evaluation

The brains from the animals treated with DMF for 7 and 25 days, as well as their respective controls were used for histological evaluation. The animals were sacrificed under deep anesthesia by intracardial perfusion with phosphate-buffered saline (PBS). Brains were removed and the hemispheres were cut sagittally in the midline. Both hemispheres together with liver and spleen were fixed in 4% (w/w) paraformaldehyde (PFA) solved in PBS overnight before embedding in paraffin. Immunohistochemistry was performed using a biotin–streptavidin peroxidase technique (DAKO, K5001) and an automated immunostainer (Autostainer Link 48, DAKO). For better antigen retrieval, sections were pretreated with citrate buffer (pH 6) for 40 min. in a steamer. After deparaffinization, intrinsic peroxidase was blocked by incubation with DAKO REAL™ Peroxidase Blocking solution (DAKO, S2023) for 5 min. All antibodies were diluted in DAKO REAL™ Antibody Diluent (DAKO, S0809). Sections were incubated with the following primary antibodies: rabbit anti-NogoA (1:200; Millipore, AB5664P), rabbit anti-Iba1 (1:500; Wako, 0199-19741), and mouse anti Nrf-2 (1:5000, Abcam, ab89443) for 30 min at RT. Sections were incubated with secondary biotinylated anti-mouse, rabbit, or rat antibodies (DAKO, K5001) for 15 min at RT. Nuclei counterstain was performed using DAKO REAL™ Hematoxylin for 5 min at RT. DAKO REAL™ DAB + Chromogene (DAKO, K3468) was used as color substrate and sections were mounted with Eukitt^®^ mounting medium (O. Kindler GmbH) after dehydration. Pictures were taken using a bright light microscope (Carl Zeiss, Oberkochen, Germany) and using 20× and 40× objectives and the software Axovision (SE64 V. 4.8). The number of cells was evaluated by blind scientists and then normalized to an area of 1 mm^2^.

### Statistics and data analysis

All results are presented as mean ± SEM. Statistical significance was analyzed in SPSS (IBM) using one-way ANOVA or two-way factorial ANOVA in case of multiple comparisons, followed by Newman–Keuls or Bonferroni post hoc test. Data were analyzed using commercial (field potentials: Clampfit, Molecular Devices; VSD: Neuroplex, RedshirtImaging; PEAK/Move, Meuth IT Consulting, Münster, Germany) and custom-made software routines (MATLAB, MathWorks). Behavioral testing was conducted and analyzed using the Noldus Ethovision System. ImageJ (National Institute of Health, Bethesda, USA) was used for histological image processing and analysis. Statistica (Statsoft, USA) and Graphpad (Prism 5, GraphPad) were used for data presentation. Graphs and figures were prepared using Origin, GraphPad, and Coreldraw X6.

## Results

### In vitro consequences of de- and remyelination on neuronal network functions

**Fig. 4:**
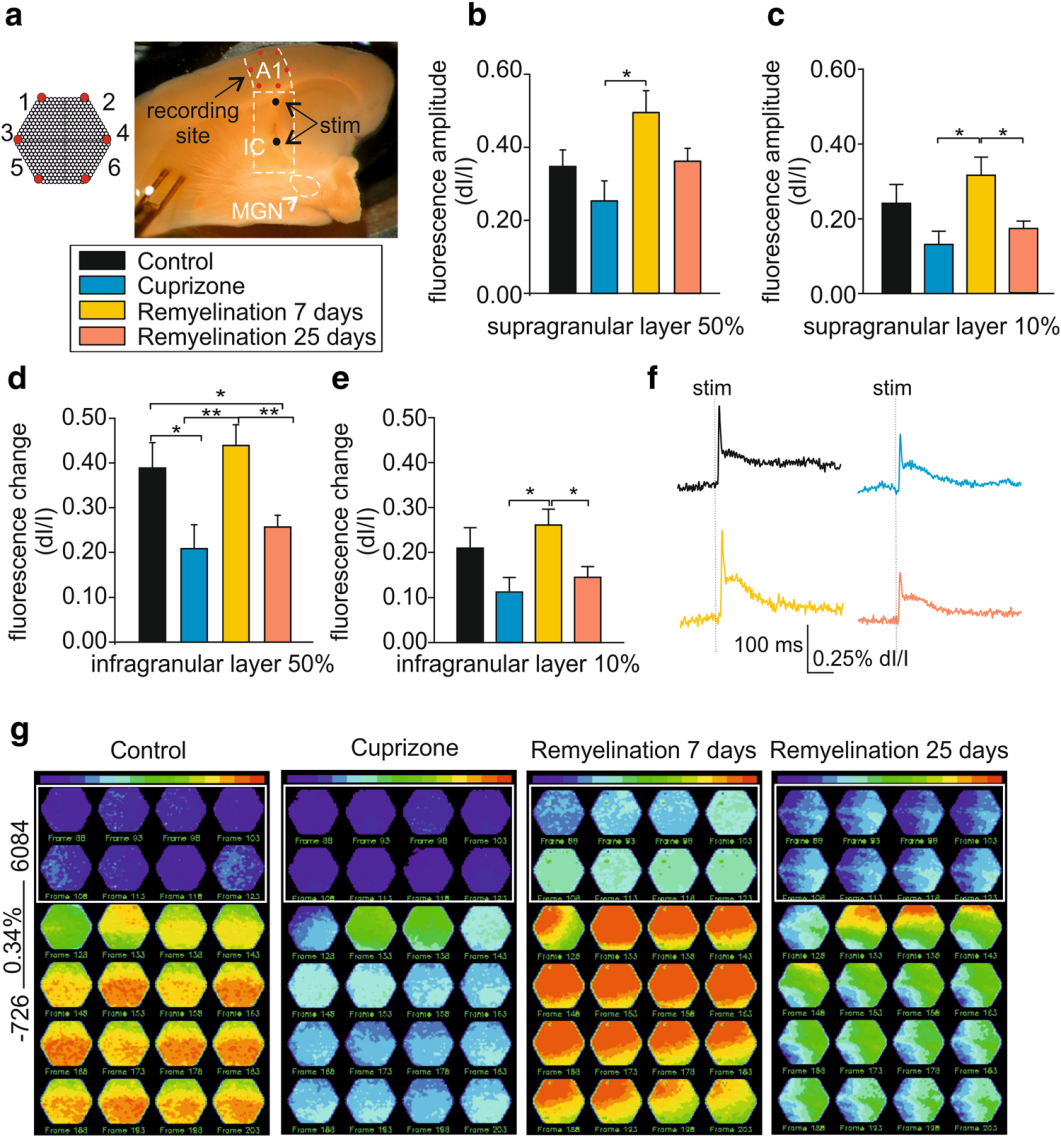
Demyelination alters neuronal network properties in the mouse thalamocortical auditory pathway *in vitro*. **a** Micrograph shows the preserved auditory thalamocortical pathway in an acute brain slice including MGN, IC, and A1. Electrical stimulation (stim) was performed at short and long distances (black dots) from A1 and simultaneous electrical and optical recordings were performed. Note the schematic representation of the VSD photodiode array (red hexagon, **a**). **b, c** Bar graphs showing the fractional fluorescence change in response to electrical stimulation of 50% (**b**) and 10% (**c**) intensity for all experimental groups in the supragranular layer. **d, e** Bar graphs showing the fractional fluorescence changes in response to electrical stimulation of 50% (**d**) and 10% (**e**) intensity for all experimental groups in the infragranular layer. **f** Example traces showing single exemplary diode traces for all experimental groups in response to 50% SI. **g** Fluorescence maps representing the spatiotemporal propagation of the VSD signal in A1 in response to electrical stimulation (50%). All experimental groups were normalized to control to appreciate the differences in fluorescence intensity, as shown by the raw diode example traces (control = 0.34% of fluorescence change)

To assess how demyelination affects the spread and dynamic properties of neuronal network activity, we investigated acute brain slices preserving the bulk of auditory thalamocortical axonal connections (Agmon and Connors 1992; Broicher et al. 2010), focusing on the primary auditory cortex (A1) as a model system. We simultaneously recorded FPs and VSD signals evoked by electrical stimulation of the internal capsule (IC; Figs. 2a, 4a). The A1 is a layered structure with tonotopic organization in which each of the six layers has specific input/output and sensory processing functions and neighboring tone frequencies are represented in adjacent cortical columns (Hackett et al. 2011; Musacchia et al. 2014). Therefore, we separately analyzed the activation of the supragranular (layers I–III) and infragranular layers (layers IV–VI). The previous studies showed a hierarchical activation pattern in many cortical areas, including A1 (Musacchia et al. 2014), which starts in the input layer IV, propagates to the supragranular layer, and then spreads to the infragranular layers. After information processing, the latter represents the output station for cortical activity to the thalamus (Barbour and Callaway 2008; Broicher et al. 2010) and we will refer mainly to the infragranular layer in the following.

Under control conditions, electrical stimulation in the IC (50%, ~ 500 µV) evoked complex FP responses that were larger in amplitude in the supragranular layer (1.77 ± 0.56 mV, *n* = 16; Fig. 2b, black bar) compared to the infragranular layer (1.69 ± 0.51 mV; *n* = 16; Fig. 2d, black bar). Following cuprizone treatment, FP amplitudes were significantly reduced in all cortical layers (supragranular: 0.74 ± 0.12 mV; infragranular: 0.68 ± 0.12 mV; *n* = 11; Fig. 2b, d, respectively, blue bars). After 7 days of remyelination, amplitudes significantly increased again even exceeding control levels (supragranular: 2.43 ± 0.23 mV; infragranular: 2.10 ± 0.45 mV; *n* = 13; Fig. 2b, d, respectively, yellow bars), thereby indicating hyperexcitability in response to electrical stimulation (Fig, 2f, yellow trace). Control-like values were reached again after 25 days of remyelination in the infragranular (1.16 ± 0.16 mV; *n* = 13; Fig. 2b, magenta bars; factorial ANOVA *F*(3, 36) = 12.95; Newman–Keuls post hoc test: cuprizone vs. control, *p* < 0.05; control vs. 7-day remyelination, *p* < 0.01; cuprizone vs. 7-day remyelination, *p* < 0.001; 25- vs. 7-day remyelination, *p* < 0.001; Fig. 2b), but not in the supragranular layer. Similar results were obtained with lower stimulation intensity (SI; 10%; Fig. 2c, e).

Construction of input/output curves representing the relationship between SI and the amplitude of the evoked FPs revealed saturating responses (Fig. 3a). To further characterize FP properties, we assessed the number of stimulations triggering population spikes (i.e., the summed action potentials (APs) of many neurons; indicated by the arrows in Fig. 3b; see “Material and methods” and Supplementary Fig. 1b) upon electrical stimulation. While FPs with 1–2 population spikes were regularly evoked under control conditions, the number of stimuli which triggered population spikes was reduced following cuprizone treatment (100% SI: 23.1 ± 8.4% vs. 58.3 ± 10.2%, respectively; 50% SI: 19.2 ± 7.9% vs. 41.7 ± 10.2%, respectively; factorial ANOVA, *F*(3,101) = 3.8; *p* = 0.013; Newman–Keuls post hoc test: 100% SI: *p* < 0.05 cuprizone vs. control, 7-day and 25-day remyelination; Fig. 3b, c). The percentage of responses with population spikes remained low after 25 days of remyelination (100% SI: 50 ± 10%; 50% SI: 38.5 ± 9.7%; Fig. 3b, c) for all the analyzed intensities. In contrast, increased excitability was observed after 7 days of remyelination (100% SI: 65.4 ± 9.5% and 50% SI: 57.7 ± 9.9%; *n* = 13) as shown by the example traces (yellow traces, Fig. 3b). These findings indicate that the degree of synchronization necessary to evoke a summed action potential in cortical neurons varies during recovery from demyelination and is permanently compromised.

### General myelin loss and remyelination alter spatiotemporal pattern of activity in A1

Taking advantage of VSD imaging, we recorded distinct changes of cortical activity with high spatiotemporal resolution as fractional changes of fluorescence intensity (Jin et al. 2002) using a photodiode array (see schematic representation in Fig. 4a). Cuprizone treatment significantly reduced the amplitude of changes in fluorescence intensity following electric stimulation (50% SI) in the supra- (control, 0.38 ± 0.05% d*I*/*I, n* = 16; cuprizone, 0.25 ± 0.05% d*I*/*I; n* = 11; Fig. 4b) and infragranular layer in comparison to controls (control, 0.34 ± 0.06% d*I*/*I*; cuprizone, 0.21 ± 0.05% d*I*/*I*; average of six diodes per group; Fig. 4d, f). After 7 days of remyelination, there was a strong increase in the response in all cortical layers indicating a strong hyperexcitability at this time point (supragranular, 0.49 ± 0.05% d*I*/*I*; infragranular, 0.44 ± 0.04% d*I*/*I, n* = 13; factorial ANOVA *F*(3, 32) = 6.2; Newman–Keuls post hoc test: cuprizone vs. control, *p* < 0.05; control vs. 25-day remyelination, *p* < 0.05; cuprizone vs. 7-day remyelination, *p* < 0.01; 7- vs. 25-day remyelination, *p* < 0.01; Fig. 4d). To assess the spatial expansion of optical signals, overlays of color-coded fluorescence-activity maps and bright-field images of the slices were constructed. The upper and lower parts of the hexagon (Fig. 4g) anatomically covered the supra- and the infragranular layer, respectively. Slices from untreated mice revealed a typical spatiotemporal pattern of activity for A1. First, the supragranular layer was transiently excited, followed by more sustained activity in the infragranular layer. Fluorescence-activity maps (normalized to the control scale of 0.34% d*I*/*I*) obtained from cuprizone-treated animals revealed low-intensity optical signals (with a preponderance of blue) compared with control conditions (Fig. 4g), suggesting decreased activity of the network or a failure to correctly convey excitation (Zendedel et al. 2013) and loss of the typical spreading pattern. Fluorescence-activity maps obtained after 7 days of remyelination showed increased stimulus-induced excitation in most cortical layers (Fig. 4g). After 25 (Fig. 4g, last panel) and 45 (data not shown) days of remyelination, optical signal intensities were lower than controls with a changed spatiotemporal pattern of activity. Analyses of recordings obtained with a lower SI revealed qualitatively comparable results in both the supra- and the infragranular layer (10% SI; Fig. 4c, e) corroborating the results observed by field potential analysis.

**Fig. 5:**
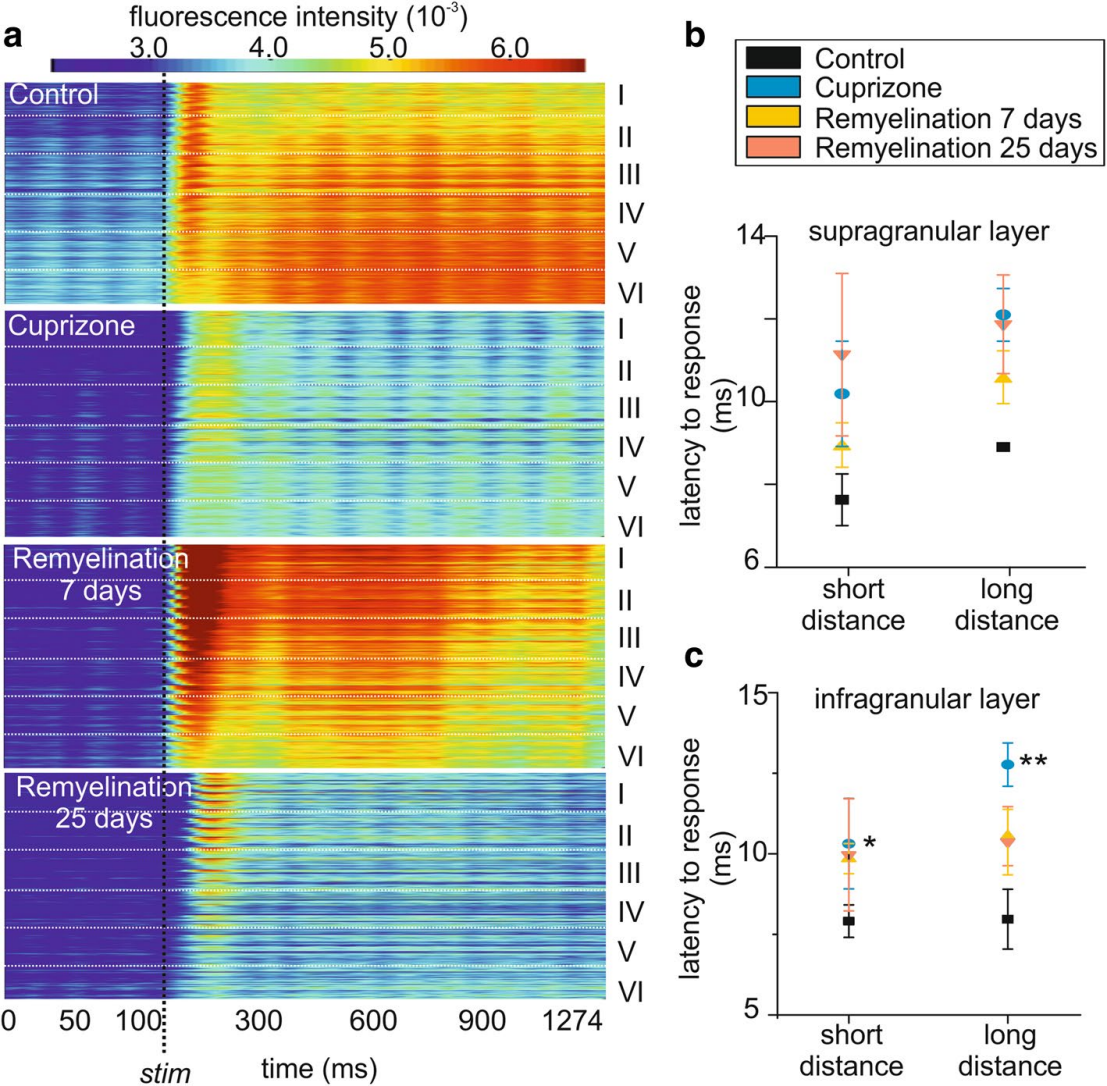
Demyelination altered the latency to response in all cortical layers. **a** Schematic representation of the spatiotemporal propagation of the evoked stimulus (*x*-axis = time in ms, *y*-axis = A1 layers) in response to electrical stimulation which is indicated by the vertical dashed line. **b, c** Response latency to electric stimulation depends on the position of the stimulation electrode: short and long distances from the cortical recording site (*n* = 6 and *n* = 7, respectively) in the supra (**b**) and infragranular layer (**c**). The latency significantly increased in cuprizone-treated animals and it is not restored during the early and late remyelination phase. *p < 0.05, **p < 0.01

To appreciate the cortical activity propagation following the electrical stimulation and the onset of the response in a more intuitive way, the signals recorded as hexagonal fluorescence maps using the photodiode array were reorganized in a new heat map taking cortical layers into account (Fig. 5a). Here, the temporal patterns of intensity changes (time is on the *x-*axes, Fig. 5a) resulting from the average of the cortical activity of five animals per experimental group are shown to propagate along the different cortical layers which are schematically represented on the *y*-axis (Fig. 5a). This data representation further remarks the altered spatial spreading of activity over time in the different experimental groups following electrical stimulation (the latter is indicated by the vertical dashed line; Fig. 5a). Moreover, from the same graph, it is possible to observe a delayed response onset in the cuprizone-treated animals in comparison to controls as indicated by the delayed appearance of warm colors after stimulation. Given that one of the first consequences of myelin loss is known to be slowing of stimulus conductance (Crawford et al. 2009b) and that in the auditory cortex timing is a prerequisite for the discrimination of auditory stimuli (Dahmen et al. 2008), we determined the response latency to stimulation in the following. In detail, latency was defined as time occurring between the electrical stimulus and the peak of the optical response in the infragranular layer of A1 by varying the position of the stimulation electrode in the IC containing the thalamocortical fiber tract. Electrical stimulation (50% SI) was performed in two distinct positions within the fiber tract: one rostral (short distance) and one caudal (long distance) to the cortex (microphotograph in Fig. 4a). In control conditions, the latency did not vary significantly, depending on the position of the stimulation electrode both in the supra- (control: short, 7.6 ± 0.5 ms, *n* = 4; long, 8.9 ± 0.0 s, *n* = 4; Fig. 5b, black squares) and infragranular layers (control: short, 8.0 ± 0.4 s; long, 8.1 ± 0.8 s; Fig. 5c, black squares), while the stimulation position had a significant effect in the infragranular layer in the absence of myelin (cuprizone: short, 10.2 ± 1.3 ms, *n* = 4; long, 12.4 ± 0.6 ms, *n* = 4; two-way ANOVA, effect of the treatment: *F*(1,28) = 9.19, *p* = 0.0012; Bonferroni post hoc test: cuprizone-long vs. control-long, *p* < 0.01; Fig. 5c, blue circles). Remyelination partly rescued the cuprizone effect in the supragranular layer, but latencies did not reach control values at any of the remyelination states (7­day remyelination: short, 9.8 ± 0.4 ms, *n* = 6; long, 10.4 ± 0.8 ms, *n* = 6, yellow triangles; 25-day remyelination: short, 10.2 ± 0.9 ms, *n* = 6; long, 11.2 ± 1.6 ms, *n* = 7; two-way ANOVA, *F*(1,26) = 2.53, *p* = 0.103; Fig. 5c).

### Dimethyl fumarate improved neuronal network activity and ameliorated behavioral deficits induced during demyelination

**Fig. 6:**
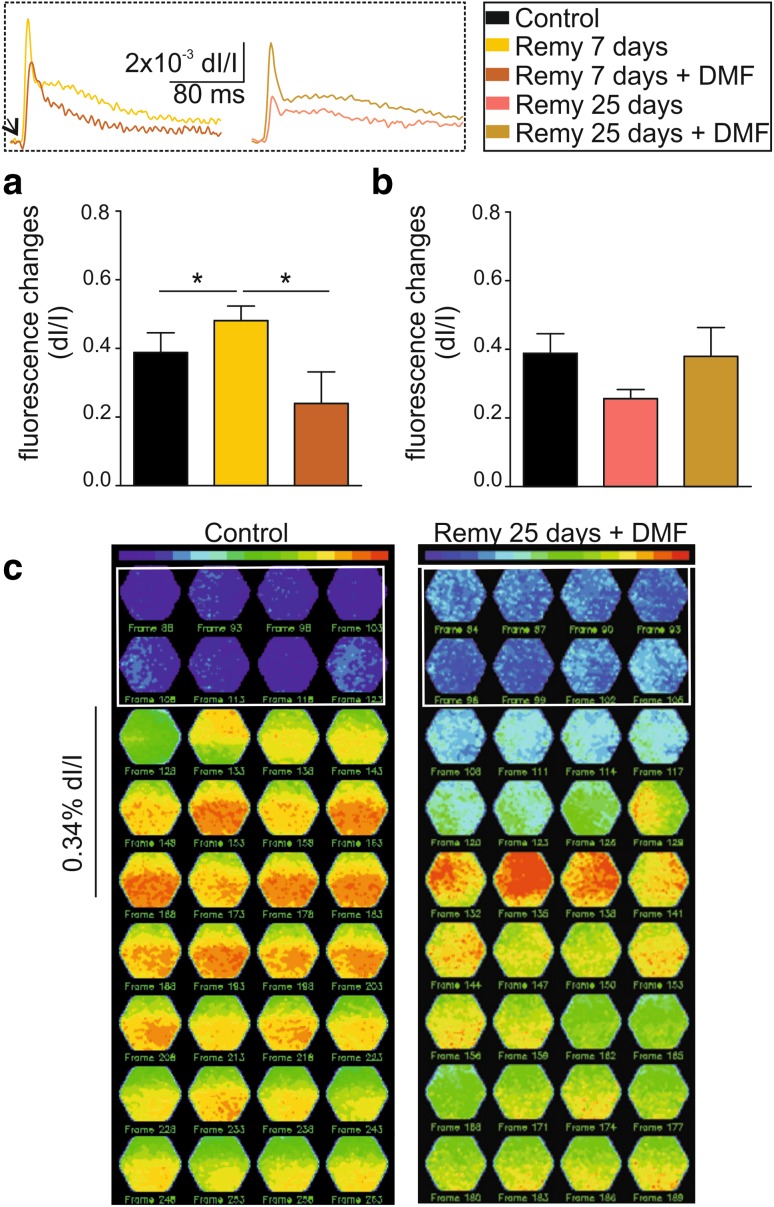
DMF treatment improves neuronal network functionality in vitro. **a** Bar graphs showing the fractional fluorescence changes in response to electrical stimulation of 50% intensity in control animals (black bars) and after 7 days of remyelination with and without DMF treatment (dark orange and yellow bars, respectively). The two left superimposed exemplary traces in the inset show the difference in amplitude between these two groups. **b** Bar graphs showing the fractional fluorescence changes in response to electrical stimulation of 50% intensity in control animals (black bars) and after 25 days of remyelination with and without DMF treatment (ocher and pink, respectively). The two right superimposed exemplary traces in the inset show the difference in amplitude between these two groups. **c** Fluorescence maps showing the spatiotemporal pattern of stimulus propagation in control 25 day DMF-treated animals. *p < *0.05*

Given the abnormal neuronal activity responses observed at 7 days of remyelination, we tried to target this time point as potential therapeutic window by treating the animals with the cytoprotective compound dimethyl fumarate (DMF; Bomprezzi 2015). After cuprizone treatment, animals were divided into two groups and treated with DMF for 7 and 25 days; their brains were then investigated ex vivo (schematic experimental outline in Fig. 1). The analysis of optical signals showed that already after 7 days of treatment with DMF upon cuprizone diet cessation, the hyperexcitability that characterized the untreated animals at the same time point was significantly reduced (one-way ANOVA, *F*(2,20) = 3.89, *p* = 0.039; pairwise comparison control and DMF 7 days vs. remyelination 7 day: *p* < 0.05; Fig. 6a, yellow bars). In the same way, animals treated with DMF during the 25 days of remyelination showed increased neuronal cortical activity in response to electrical stimulation compared to the untreated animals at the same time point (infragranular: 0.38 ± 0.08%; Fig. 6b). Analysis of the field potential responses in the infragranular layer of the A1 qualitatively confirmed these results by showing responses very similar to control values at 7 and 25 days of DMF treatment (Supplementary Fig. 2a and b). However, due to the larger variability of field potential responses compared VSD measurements, no significant differences were obtained for the different recording conditions. Moreover, further constructing heat-fluorescence maps from DMF-treated animals revealed a normalized spatiotemporal activation. In detail, a response in the supragranular layer followed by propagation into the lower layers was observed, as shown before for the control animals (Fig. 6c).

**Fig. 7:**
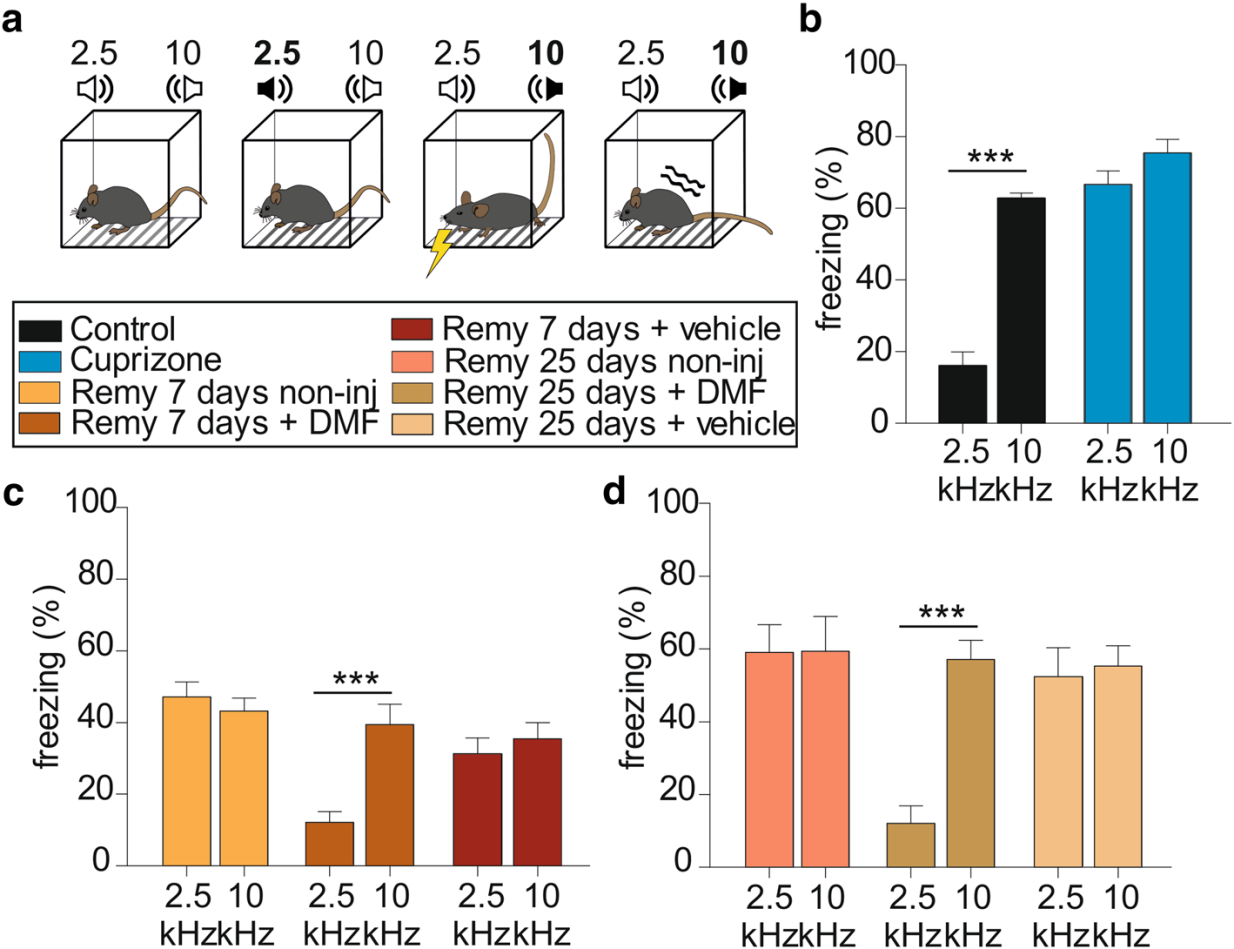
DMF administration ameliorates the auditory cortical functioning in vivo. **a** Schematic representation of the Pavlovian fear conditioning paradigm: animals were presented with the two tones of 2.5-and 10-kHz frequency, respectively. The 10-kHz tone was associated to a mild foot-shock (conditioned stimulus—indicated by the yellow flash sign in the third box from the left). The animals were adapted to the two tones on day 1 by random presentation of the tone twice per day. During the second day, the 10-kHz frequency was associated with a foot-shock. The third day, animals were randomly presented with the two tones, without any foot-shock and the reaction to the conditioned stimulus was quantified as immobility behavior (freezing—indicated by the wave sign). **b** Bar graphs showing the percentage of freezing in response to 2.5 and 10 kHz in the control group (black bars) and in cuprizone-treated animals (blue bars). **c** Bar graphs showing the percentage of freezing in response to 2.5 and 10 kHz in animals treated with DMF for 7 days after remyelination starts (dark orange bars) in comparison to vehicle-treated and non-injected animals (dark red and yellow bars, respectively). **d** Bar graphs showing the percentage of freezing in response to 2.5 and 10 kHz in animals treated with DMF for 25 days after remyelination starts (olive green bars) in comparison to vehicle-treated and non-injected animals (light brown and pink bars, respectively). ***p < *0.001, 2.5 vs. 10 kHz*

To determine whether the normalization of network activity is reflected in the behavior, we used a Pavlovian fear conditioning paradigm that was previously proven adequate for monitoring cuprizone-induced changes in learned behavior (Cerina et al. 2017). Animals were challenged with the presentation of two different tones, 2.5 and 10 kHz (schematic representation in Fig. 7a). The latter tone was associated with an electrical foot-shock and freezing in response to the presentation of the conditioned tone was taken as a read-out. Successfully conditioned animals showed low freezing for the 2.5 kHz frequency (16.1 ± 3.8%) compared to the conditioned tone (62.8 ± 1.4%; *n* = 12; Fig. 7b). Indeed, mixed design factorial ANOVA revealed a significant effect of the frequency as variable: *F*(1,55) = 78.5, *p* < 0.0001 and pairwise comparison: *p* < 0.001. Moreover, a significant interaction between the effect of frequency and group/treatment was also observed (*F*(7,55) = 17.5, *p* < 0.0001). Following cuprizone treatment, the animals lost their discrimination abilities, which were reflected by a similar percentage of freezing upon presentation of both frequencies (2.5 kHz: 66.7 ± 3.6% and 10 kHz: 75.5 ± 3.7%; *p* = 0.07: blue bars; *nn* = 10; Fig. 7b). The same test performed 7 days after the start of remyelination in non-injected (2.5 kHz: 47.2 ± 4.2% and 10 kHz: 43.2 ± 3.5%; *p* = 0.53; yellow bars; *n* = 5; Fig. 7c) and vehicle-injected animals (2.5 kHz: 31.3 ± 4.4% and 10 kHz: 35.5 ± 4.4%; *p* = 0.5; red bars; *n* = 5; Fig. 7c) showed a similar result with high percentage of freezing in response to both tones. Interestingly, after 7 days of daily injections with DMF, the animals showed freezing only in response to the relevant tone in a control-like manner (2.5 kHz: 12.1 ± 2.9% and 10 kHz: 39.5 ± 5.6%; dark orange bars; *p* < 0.001; *n* = 9; Fig. 7b). Discrimination loss persisted in non-injected and vehicle-treated animals until 25 days of remyelination, while the treatment with DMF normalized the behavior (2.5 kHz: 12.1 ± 4.7% and 10 kHz: 57 ± 5.4%; *p* < 0.001; olive green bars; *n* = 10; Fig. 7d). Furthermore, the treatment with DMF did not alter the anxiety-like behavior and locomotor activity of the animals (Supplementary Fig. 3). These findings indicate that DMF treatment can rescue the behavioral phenotype of general de- and remyelination.

These results suggest a beneficial effect of DMF treatment on neuronal network function and histological staining was used to assess potential mechanisms in the following. Using the oligodendrocyte-specific marker NogoA, an increase in the number of positive cells was found during remyelination; however, there was no significant difference between DMF-treated and untreated mice at 7 days of remyelination (end of cuprizone treatment: 256.3 ± 23.89 cells/mm^2^; Remy 7 day non-injected: 432.9 ± 40.51 cells/mm^2^; Remy 7 day vehicle: 470.9 ± 51.17 cells/mm^2^ and Remy 7 days DMF: 501.3 ± 30.11 cells/mm^2^; one-way ANOVA, *F*(7,30) = 4.79, *p* = 0.001; Tukey’s post hoc test: Remy 7-day vehicle and Remy 7-day DMF vs. Cuprizone, *p* < 0.05 and *p* < 0.01, respectively; Supplementary Fig. 4a and d). At 25 days of remyelination, numbers of NogoA-positive cells were slightly further increased, but still there were no differences between treated and untreated animals (*p* = 0.87; Supplementary Fig. 4a and d).

Next, we used Iba-1 as marker for microglia and we counted the activated cells which show increased size and amoeboid morphology in comparison to non-activated cells (Gudi et al. 2014; Supplementary Fig. 4b and e). While the state of complete demyelination was accompanied by a strong increase of activated microglia (one-way ANOVA, *F*(7,31) = 11.82, *p* < 0.0001; Tukey’s post hoc test: cuprizone vs. control: *p* < 0.0001), their numbers decreased again with ongoing remyelination. DMF treatment did not change this time-dependent decrease, although the decline was less pronounced at 7 days of remyelination for the DMF- and vehicle-treated groups.

When the expression and subcellular localization of Nrf-2 was analyzed in the following, we observed significant effects of DMF treatment which were in line with the previous findings (Ramsey et al. 2007). A mixed design factorial ANOVA revealed that the de- and remyelination paradigm (effect of the treatment: *F*(1,51) = 137.4, *p* < 0.0001) significantly influence the subcellular distribution of Nrf-2 in A1 (effect of the interaction between the variables: *F*(7,51) = 15.30, *p* < 0.0001). In detail, under control conditions, the transcription factor was mainly detected in the nucleus (where it regulates the gene expression of anti-oxidative proteins) rather than the cytoplasm (518.9 ± 72 cells/mm^2^ and 208.86 ± 18.27 cells/mm^2^, respectively; *p* < 0.05; Supplementary Fig. 4c and f). Cuprizone treatment reversed this distribution and the number of cells positive for Nrf-2 only in the nucleus was significantly decreased compared to control (255.69 ± 47.6 cells/mm^2^; *p* < 0.05; Supplementary Fig. 4c and f), while the number of cells showing only a cytoplasmatic localization was significantly increased (496.83 ± 14.03 cells/mm^2^, *p* < 0.05; Supplementary Fig. 4c and f). Spontaneous remyelination at 7 days after removal of cuprizone from the diet was associated with a reduction of the number of cytoplasmatic Nrf-2 cells both in non-injected (231.01 ± 59.61 cells/mm^2^; *p* < 0.05 vs. cuprizone) and vehicle-treated animals (255.69 ± 31.87 cells/mm^2^; *p* < 0.05 vs. cuprizone). At the same time, the number of cells expressing Nrf-2 in the nucleus increased, although numbers were not significantly different when cuprizone and both in non-injected (409.28 ± 11.16 cells/mm^2^) and vehicle-treated animals (420.88 ± 39.14 cells/mm^2^) were compared. Importantly, the group treated with DMF for 7 days of remyelination showed a significant increase of the nuclear Nrf-2-positive cells in comparison to the cuprizone group (610.13 ± 84.1 cells/mm^2^; *p* < 0.0001 vs. cuprizone nucleus; Supplementary Fig. 4c and f). At 25 days of remyelination, non-injected (544.30 ± 57.78 cells/mm^2^; *p* < 0.01 vs. cuprizone nucleus), vehicle-injected (677.21 ± 84.10 cells/mm^2^; *p* < 0.0001 vs. cuprizone nucleus), and DMF-treated (740.50 ± 23.39 cells/mm^2^; *p* < 0.0001 vs. cuprizone nucleus: Supplementary Fig. 4c and f) groups showed significantly increased numbers of nuclear Nrf-2 cells in comparison to cuprizone-treated animals but no differences with respect to each other. This held true also for the number of cells showing cytoplasmatic localization only.

## Discussion

Here, we assessed the effect of de- and remyelination on FP responses and the spread of neuronal activity in thalamocortical slices following electrical stimulation of the IC in the cuprizone model. Our results show that myelin loss profoundly affects spatiotemporal patterns of neuronal activation and signal propagation in the mouse auditory cortex. In addition, these changes are reflected by a loss of auditory discrimination in vivo, which persists during the early and late phases of remyelination. Importantly, therapeutic administration of the cytoprotective compound DMF resulted in the improvement of cortical network functionality following demyelination.

### Myelin loss and gain affect auditory cortical neuronal network spatiotemporal functionality in acute brain slices and in freely behaving animals

Previously, we have shown that the changes induced by demyelination in the auditory cortex trigger a profound impairment in the ability to discriminate between auditory frequencies in vivo (Cerina et al. 2017). Interestingly, this functional deficit persisted upon spontaneous remyelination, suggesting that the loss of myelin was not the sole cause of malfunction. Indeed, we show here that the impairment in auditory discrimination is associated with a profound and persistent alteration in the cortical auditory neuronal network activity in vitro. Upon demyelination, VSD and FP activity was dampened, and associated with a reduction of the myelin content in white matter fiber tracts. Because of slowed conduction velocities, propagation of stimuli between different neuronal populations may be delayed, and in the case of axon degeneration, may even become lost, thereby altering information processing (Crawford et al. 2009a, b). Indeed, here, we observed increased response latencies in the auditory cortex upon electrical stimulation of the IC. In addition, during the early phase of remyelination, we observed neuronal hyperexcitability that was characterized by increased response amplitudes, an increased occurrence of population spikes, and an altered spread of activity following electrical stimulation in all cortical layers at any of the stimulation intensities. This increased excitability was only transitory, because, at a later stage of remyelination (25 days), neuronal response levels were lower than controls, and myelin gain did not restore neuronal network activity. While at both time points were response latencies declined again, they did not reach control-like values although remyelination had occurred. The reasons may be manifold and seem to be only partly attributed to the myelin loss/gain process. It has been shown that prolonged cuprizone treatment is associated with a decrease in axon diameter (Wu et al. 2008; Cate et al. 2010). Both demyelination and decrease in axon diameter will reduce axonal conduction velocity. Moreover, disruption of energy production, redistribution ion channels, and axonal degeneration and loss may alter information processing and excitability (Crawford et al. 2009a, b). In this respect, transient changes in excitability were already suggested to be the initial event leading to long-lasting deficits and neurodegeneration following loss of myelin in other disease models (Sutor et al. 2000; Busche et al. 2008; Ghaffarian et al. 2016). In fact, it is known that myelin synthetized following demyelination, especially when the synthesis is triggered by removal of cuprizone treatment, is characterized by regionalization (Brousse et al. 2015), thinner sheaths (Zendedel et al. 2013), altered distribution of Nodes of Ranvier (Crawford et al. 2009b), and reduced tightness between the wrapped sheathing (Stidworthy et al. 2006; Praet et al. 2014). However, it is also known that such characteristics do not hinder neuronal functional improvement when only white matter regions are affected (Cerina et al. 2017). Therefore, it is reasonable to hypothesize that neuronal activity and latency were profoundly and long-lastingly affected (Crawford et al. 2009b; Cerina et al. 2017). A similar scenario was already suggested by other groups reporting reorganization of cortical layers and cortical networks following re-arrangements of different cortical cell types under a number of pathophysiological conditions (Sutor et al. 2000; Markoullis et al. 2012b; Saji et al. 2013). Thus, loss of myelin may compromise neuronal network architecture as well as functionality. Furthermore, neuronal reorganization may not only involve cortical columns in a vertical orientation, but also short- and long-range connections between cortical regions and hemispheres (Hübner et al. 2017). Accordingly, our findings show a persistent change of neuronal network activity emerging from the analysis of the spatiotemporal patterns of stimulus propagation, possibly reflecting the reorganization of tonotopic maps in A1. The normal activation pattern of A1 upon electrical stimulation (Broicher et al. 2010) starts with activation of layer IV, which together with layer III is described as the input layer (Linden and Schreiner 2003; Barbour and Callaway 2008), and is subsequently propagating to the supragranular layers. Finally, the stimulus spreads to the infragranular layers where it is redirected to subcortical regions (Kubota et al. 1999; Sherman 2012). Immediately following demyelination, this strict pattern in the spatial spreading of activity was completely absent as the neuronal activation was significantly dampened compared to control. During the different phases of remyelination, altered activity spreading was observed in A1. During the early phase of remyelination (7 days), a clear time- and space-dependent activation of the supra- and infragranular layers was absent because of an extensive high-amplitude response to electrical stimulation. Similar conditions of hyperexcitability were also observed in other animal models of MS (Meuth et al. 2009; Hamada and Kole 2015; Luchtman et al. 2016). While this increased excitability was transitory, the spatiotemporal pattern of activation seems to be persistently altered even after full remyelination (25 days; Cerina et al. 2017). Importantly, altered functional A1 activity was also observed in the behavior of the animals. When challenged with a sensory conditioning task involving higher brain function, the animals failed in discriminating different auditory stimuli both after demyelination and at different stages of remyelination.

### The molecular substrates for discrimination deficits

Taken together, our results suggest that proper network function with a distinct sequence, speed, and synchronization of information processing is necessary to sustain tonotopic auditory maps in the cortex and cognition-related behavior in vivo. It has been suggested previously that altered activity spreading very likely indicates altered cortical sensory processing (Linden and Schreiner 2003; Broicher et al. 2010). From a physiological point of view, well-functioning spatiotemporal stimulus propagation is important for discriminating and extracting increasingly abstract features of incoming information by different cortical regions (Gavornik and Bear 2014; Groh et al. 2014). The maintenance of cortical hierarchical structures is essential, since pharmacological silencing of connections between cortical layers results in basal reorganization involving neighboring cortices at multiple sites (Kaur et al. 2005). In a similar manner, altered cortical connections were recently described as common hallmarks in various neurological diseases like MS and Alzheimer’s disease, both in patients and animal models (Gamboa et al. 2014; Spence et al. 2014; Tambalo et al. 2015). Alterations were associated with cognitive and locomotor deficits (Markoullis et al. 2012a; Gamboa et al. 2014), thus supporting the notion that damage to sensory areas affects the performance of higher brain functions. Since MS is characterized by numerous pathologies, dissecting underlying mechanisms is difficult (Zoupi et al. 2013; Mighdoll et al. 2014). Nevertheless, increased excitability following demyelination was attributed to the altered expression and distribution of specific ion channels such as Cav1.2, Nav1.3, and Kv7.3 that are exposed on the axon surface after myelin loss and are re-distributed along the neuronal body resulting in changed physiological functionality (Crawford et al. 2009b; Hamada and Kole 2015). Indeed, alteration of ion channels is a hallmark which would support both our findings of altered neuronal network functionality and excitability, since all of the above-mentioned channels are involved in regulating neuronal excitability (Ehling et al. 2011). In a similar manner, recent evidence showed an altered distribution and number of interneurons in motor cortex (Falco et al. 2014), and a misbalance between GABAergic and/or glutamatergic functioning (Olechowski et al. 2010; Potter et al. 2016) in experimental autoimmune encephalomyelitis (EAE) which were responsible for altered excitability in motor and somatosensory cortex, thus pointing to common hallmarks in different models of MS.

### Therapeutic intervention with DMF during remyelination improves neuronal network functionality in vitro and in vivo

Since our results pointed to persistently altered neuronal network functions in A1 with changes in excitability and stimulus propagation patterns during the early phase of demyelination, we performed a “therapeutic” pharmacological intervention in our animal model using the well-known cytoprotective compound DMF (Tambalo et al. 2015). The treatment with DMF was started at the end of the demyelinating diet taking into account that, after removal of cuprizone, spontaneous remyelination starts (Matsushima and Morell 2001; Skripuletz et al. 2008). To gain a temporal profile of the DMF effect and identify an optimal time period for potential therapeutic intervention, we analyzed the two points in time, namely 7 and 25 days of remyelination which we have characterized before and assessed cognitive impairments in vivo and neuronal network responses ex vivo. Both 7 and 25 days of treatment normalized VSD and FP responses in vitro to values similar to controls. Importantly, the treatment after 7 days reduced hyperexcitability, which was associated with the early remyelination, suggesting that targeting this period leads to amelioration of neuronal network functionality in vitro. The normalized spatiotemporal spreading of activity seems to be associated with a reconstituted discrimination ability of the primary auditory cortex. Animals treated with DMF for 7 and 25 days during remyelination showed normal-frequency discrimination and a phenotype rescue. When challenged with the presentation of two different frequencies, mice were able to distinguish between them and reacted with the properly conditioned response, thus suggesting that neuroprotection during the remyelination phase might be a good strategy to pursue. However, it is unclear whether DMF has sole neuronal targets. DMF has proven to be very well accepted by patients due to its route of administration (oral), and was shown to be effective in patients presenting with relapsing-remitting forms of MS (Kappos et al. 2008; Bomprezzi 2015). However, various mechanisms of action of DMF were described both for patients and in animal models. The most accredited results focused on the effect exerted as anti-oxidative stress compound (Scannevin et al. 2012). In more detail, the active DMF metabolite MMF promotes the transcription of a higher number of anti-oxidative genes by increasing the amount of the nuclear factor Nrf2, thus restricting a damage derived from potential oxidative stress (Scannevin et al. 2012; Fox et al. 2014). Here, cuprizone diet heavily reduced the number of cells expressing Nrf-2 in their nucleus while increasing the ones showing a cytoplasmic expression. Other neurodegenerative diseases were also characterized by this particular event following an external insult (Ramsey et al. 2007). In this respect, it is known that administration of the copper chelator cuprizone modifies mitochondrial morphology and functioning in mature oligodendrocytes, a condition which is often considered to occur as a consequence of activating oxidative stress mechanisms (Praet et al. 2014). This seemed to be corroborated by our findings, since following DMF treatment for 7 days of remyelination, the number of cells expressing Nrf-2 in the nucleus was significantly increased in comparison to vehicle application. Interestingly, the effect was still observed at 25 days of remyelination; however, there were no differences between untreated and DMF-treated animals. Therefore, it is reasonable to hypothesize a positive effect exerted by DMF at 7 days of remyelination, which coincided with the restoration of control-like values in the auditory cortical neuronal network and amelioration of cognitive skills. Concerning the mechanism underlying such improvement, our findings pointed to a cytoprotective effect involving neurons, although we were not able to clarify whether it was an indirect mechanism or direct mechanism. In the present study, treatment with DMF in the cuprizone model does not promote remyelination or oligodendrocyte survival, thus supporting the idea of an involvement of additional mechanisms and cell types as previously suggested (Ramsey et al. 2007; Moharregh-Khiabani et al. 2010). It is well known that DMF and its metabolite have an effect in regulating inflammation and immune cells activation (de Jong et al. 1996; Linker et al. 2011; Reick et al. 2014). Evidence showing a shift from activated pro-inflammatory cells and an increase of B cells in MS patients would support this hypothesis (Moharregh-Khiabani et al. 2010; Reick et al. 2014; Li et al. 2017). However, in the cuprizone model, major contributions of inflammation could be ruled out (Skripuletz et al. 2008, 2011; Cerina et al. 2017). This, as well as infiltration of immune cells into the CNS, is a typical feature in models of focal and general experimental autoimmune encephalitis (Pierson et al. 2012). Despite the still unknown exact mechanism of action of DMF, taking into consideration the ameliorative effects on neuronal network functionality and higher brain functions, we conclude that a further step forward in understanding the role of neuronal impairment in the pathophysiology of MS was made. In addition, further experiments will clarify if the decision to focus on some time windows for therapeutic intervention could pave the way to understand further mechanism and also to gain more insights in the mechanism of action of DMF.
